# Grape Seed Proanthocyanidin Extract Attenuates Cafeteria-Diet-Induced Liver Metabolic Disturbances in Rats: Influence of Photoperiod

**DOI:** 10.3390/ijms25147713

**Published:** 2024-07-14

**Authors:** Romina M. Rodríguez, Marina Colom-Pellicer, Julia Hernández-Baixauli, Enrique Calvo, Manuel Suárez, Anna Arola-Arnal, Cristina Torres-Fuentes, Gerard Aragonès, Miquel Mulero

**Affiliations:** 1 Nutrigenomics Research Group, Department of Biochemistry and Biotechnology, Universitat Rovira i Virgili (URV), Campus de Sescelades, 43007 Tarragona, Spain; rominamarielrodriguez@gmail.com (R.M.R.); marina.colom@urv.cat (M.C.-P.); enrique.calvo@urv.cat (E.C.); manuel.suarez@urv.cat (M.S.); anna.arola@urv.cat (A.A.-A.); cristina.torres@urv.cat (C.T.-F.); gerard.aragones@urv.cat (G.A.); 2 Laboratory of Metabolism and Obesity, Vall d’Hebron-Institut de Recerca, Universitat Autònoma de Barcelona, 08035 Barcelona, Spain; julia.hernandez@vhir.org; 3 Center of Environmental, Food and Toxicological Technology-TecnATox, Rovira i Virgili University, 43201 Reus, Spain

**Keywords:** photoperiod, circannual rhythms, obesity, cafeteria diet, liver lipidic metabolism, grape seed proanthocyanidin extract

## Abstract

This study investigated the influence of photoperiod (day length) on the efficacy of grape seed proanthocyanidin extract (GSPE) in mitigating metabolic disorders in obese rats fed a cafeteria diet. Rats were exposed to standard (L12), long (L18), or short (L6) photoperiods and treated with GSPE or vehicle. In the standard photoperiod, GSPE reduced body weight gain (50.5%), total cholesterol (37%), and triglycerides (34.8%), while increasing the expression of hepatic metabolic genes. In the long photoperiod, GSPE tended to decrease body weight gain, increased testosterone levels (68.3%), decreased liver weight (12.4%), and decreased reverse serum amino acids. In the short photoperiod, GSPE reduced glycemia (~10%) and lowered triglyceride levels (38.5%), with effects modified by diet. The standard photoperiod showed the greatest efficacy against metabolic syndrome-associated diseases. The study showed how day length affects GSPE’s benefits and underscores considering biological rhythms in metabolic disease therapies.

## 1. Introduction

Evolution has conserved biological rhythms across taxa, allowing physiological and behavioral responses to cyclic environmental changes [1,2]. Thus, adaptations to the 24 h day–night cycle (circadian rhythm) and to the 365-day seasonal cycle (circannual rhythm) allows the timing optimization of fundamental cellular and physiological processes and behaviors [3,4]. In this sense, processes such as body temperature, hormone secretion, blood pressure and immune and metabolic functions show periodic oscillations and are regulated, in part, by an innate timing system, the so-called circadian clock [5]. In mammals, the circadian timing system comprises a central pacemaker located in the suprachiasmatic nucleus of the hypothalamus, which receives the photic information captured by cells in the retina via the retinohypothalamic tract, and is able to generate, adjust, and sustain rhythms affecting whole-body physiology [6]. In addition to the central oscillator, there are peripheral clocks located in nearly every tissue and organ of the human body which can be regulated by the master clock but also show an independent physiological control in synchrony with the environment [7].

The circadian clock regulates the sleep–wake and feeding–fasting cycles by responding to light changes in our environment [8]. In fact, changes in photoperiod, resembling seasonal day length variations, have been shown to alter circadian rhythmic expression and to impair lipid metabolism in rats [9]. In addition, a photoperiod-dependent response in terms of body weight gain, eating behaviors, and lipid and glucose metabolism has been demonstrated in healthy and obese photoperiod-sensitive Fischer (F344) rats [10,11,12]. 

Moreover, several studies have demonstrated that some human patterns of natality, mortality, and illness could be influenced by the seasons [13]. Additionally, circulating levels of triglycerides, cholesterol, glucose, and insulin were found to be elevated in winter, together with a higher accumulation of body fat than in summer, contributing to an increase in cardiometabolic diseases, especially during the winter season [14,15,16].

Not only light but also food has been shown to entrain the molecular clock machinery, especially the clocks located in metabolic organs, such as the liver [17]. Therefore, metabolism and circadian rhythms are intimately and reciprocally linked [18,19]. There is increasing evidence showing that an excessive caloric intake causes disturbances in the circadian system and consequently a disruption of metabolic circadian synchrony, increasing the risk of developing metabolic disorders, including obesity, type 2 diabetes mellitus (T2DM), metabolic syndrome (MS), and non-alcoholic fatty liver disease (NAFLD) [20,21,22,23]. Due to the increasing rise of metabolic diseases, mainly caused by modern human lifestyle with constant food availability, increased intake of the Western diet, sleep deprivation, and sedentary behaviors; the discovery of new strategies to mitigate these metabolic disorders has intensified in the last years. In this regard, polyphenols, and particularly proanthocyanidins, have emerged as promising natural plant-based compounds capable of ameliorating metabolic alterations associated with MS and related diseases. Several studies have evidenced the health benefits of its consumption, protecting against cardiovascular disease, obesity, dyslipidemia, hyperglycemia, and liver damage [24]. Moreover, it has been demonstrated that grape seed proanthocyanidin extract (GSPE) is able to modulate both central and peripheral clocks not only in healthy but also in diet-induced obesity animals [25,26], suggesting a possible mechanism involved in its beneficial effects and allowing the restoration of the circadian misalignment caused by the obesogenic diet [27]. Additionally, it has been recently reported that GSPE was able to attenuate the alterations in feeding patters, locomotor activity, and serum hormones caused by an abrupt change in photoperiod in healthy and obese rats [28]. 

We have previously reported a seasonal modulation of the effects of GSPE on glucose and lipid metabolism in the liver of healthy rats [29]. However, the specific impact of seasonal variations in day length (i.e., photoperiod) on the efficacy of GSPE in modulating the hepatic circadian clock and metabolic functions in obesity remains unexplored. Hence, this study aims to evaluate the influence of photoperiods on the metabolic effects of GSPE in obese rats. To achieve this purpose, photoperiod-sensitive rats (F344) were fed a cafeteria (CAF) diet and chronically exposed to three different photoperiods (L12, L18, and L6) to mimic the day length of different seasons, and were treated either with GSPE or vehicle (VH). Rats fed a standard (STD) diet and treated with VH were used as a normal-weight control in each photoperiod. We further conducted biochemical, gene expression, serum and liver metabolomics, and hepatic lipid characterization assays to assess whether changes in photoperiod exposure can influence the molecular effect of GSPE on metabolism in obese rats.

## 2. Results

### 2.1. The Effect of GSPE on Body Weight Gain in CAF-fed Rats Differs according to the Photoperiod

**Figure 1:**
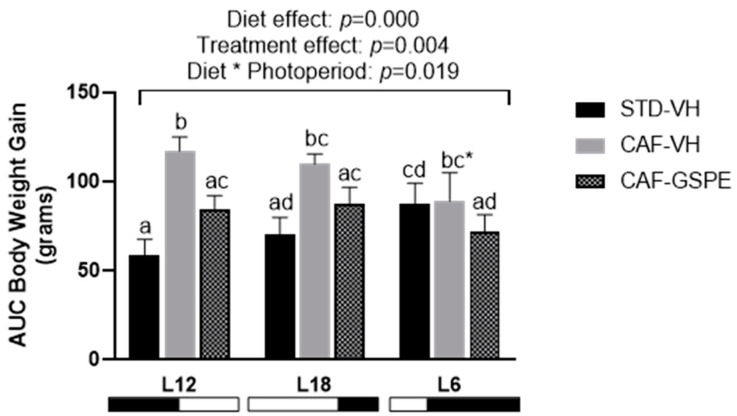
Body weight gain as area under the curve (AUC) in Fischer 344 STD and CAF-fed rats exposed to standard (12 h light:12 h dark), long (18 h light:6 h dark), or short (6 h light:18 h dark) photoperiods (*n* = 8). The results are presented as the mean ± S.E.M. One- and two-way ANOVA following LSD post hoc tests were performed to compare the values between groups, and significant differences (*p* ≤ 0.05) were represented with different letters (a, b, c, d). A * indicates tendency between L18-CAF-VH and L18-CAF-GSPE groups (*p* = 0.059).

The intake of an obesogenic diet is reflected in the body weight gain of the animals subjected to a CAF diet as it was significantly increased in rats exposed to L12 and L18, but not in L6-CAF-VH animals compared to their STD diet controls (Figure 1). This was corroborated with the results of two-way ANOVA that showed an interaction between diet and photoperiod (*p* = 0.019). The chronic consumption of GSPE reduced body weight gain in L12 animals (*p* = 0.020) and tended to lower the body weight of animals exposed to a long photoperiod (L18) (*p* = 0.059), respectively, compared to its CAF-VH, whereas this effect was not observed in L6-CAF-GSPE rats.

### 2.2. The Impact of GSPE on Serum Parameters Is Influenced by the Photoperiod in CAF-fed Rats

**Figure 2:**
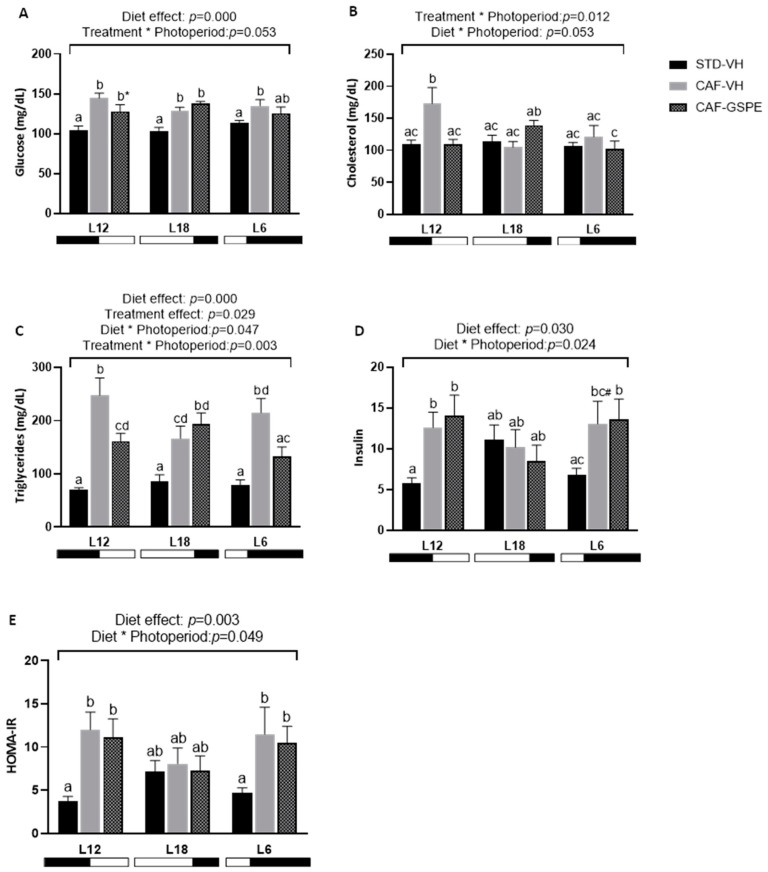
Serum parameters of Fischer 344 STD and CAF-fed rats exposed to L12, L18, or L6 photoperiods (n = 8) (**A**–**E**). The results are presented as the mean ± S.E.M. One- and two-way ANOVA following LSD post hoc tests were performed to compare the values between groups, and significant differences (*p* ≤ 0.05) were represented with different letters (a, b, c, d). A * indicates tendency between L12-CAF-VH and L12-CAF-GSPE groups (*p* = 0.053). A # indicates tendency between L6-STD-VH and L6-CAF-VH groups (*p* = 0.054).

As is shown in Figure 2, the serum levels of glucose were significantly higher in all CAF-VH groups compared to their STD, whereas the effect of GSPE treatment was only observed in the L12 photoperiod with a tendency of decreasing serum glucose levels compared to L12-CAF-VH animals (*p* = 0.053). Moreover, L12-GSPE animals also showed a significant decrease in total serum cholesterol in comparison to its CAF-VH (*p* = 0.001). Regarding serum triglycerides levels, a clear interaction between treatment and photoperiod was observed (*p* = 0.003), since triglycerides values were elevated in all CAF-VH groups but could only be lowered by GSPE treatment in animals subjected to L12 (*p* = 0.003) and L6 (*p* = 0.006) photoperiods. Insulin levels also showed an interaction between diet and photoperiod (*p* = 0.024) where CAF-VH increased or tended to increase its values in L12 (*p* = 0.023) and L6 (*p* = 0.054) photoperiods, but not in L18. Similar results were observed for HOMA-IR.

**Figure 3:**
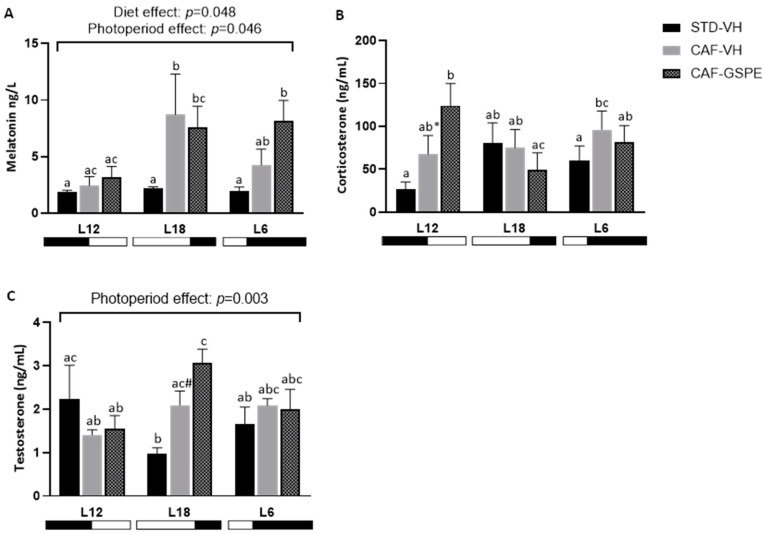
Serum hormones of Fischer 344 STD and CAF-fed rats exposed to L12, L18 or L6 photoperiods (n = 8) (**A**–**C**). The results are presented as the mean ± S.E.M. One- and two-way ANOVA following LSD post hoc tests were performed to compare the values between groups, and significant differences (*p* ≤ 0.05) were represented with different letters (a, b, c). A * indicates tendency between L12-CAF-VH and L12-CAF-GSPE groups (*p* = 0.054). A # indicates tendency between L18-CAF-VH and L18-CAF-GSPE groups (*p* = 0.055).

Serum levels of melatonin, corticosterone and testosterone were analyzed and plotted in Figure 3. In this regard, melatonin levels were found to be affected by photoperiod (*p* = 0.048) and diet (*p* = 0.046) showing an increase in L18 and L6 CAF-fed animals. Testosterone levels were also strongly influenced by photoperiods (*p* = 0.003), and animals treated with GSPE under long day conditions had higher testosterone levels compared to the lean control group (*p* = 0.001) and tended to increase compared to CAF-VH rats (*p* = 0.055). Similar results were observed in L12 conditions for serum corticosterone levels, where GSPE treatment increased or tended to increase corticosterone values compared to STD-VH (*p* = 0.001) and CAF-VH (*p* = 0.054) groups.

### 2.3. Photoperiod Significantly Influences Lipid Levels in the Livers of Obese Rats

As is shown in Table 1, liver cholesterol values were increased due to CAF-diet intake compared to the STD lean control independently of the photoperiod but were higher in L18-CAF-VH rats compared to L12 counterparts (*p* = 0.024). Furthermore, a strong influence of photoperiod was also observed in triglycerides levels (*p* = 0.001), as they raised in all CAF-VH groups compared to STD groups, but triglycerides levels of CAF-fed animals subjected to long or short photoperiod were even higher compared to L12 CAF-fed rats. Similar results were observed on phospholipids content with a tendency of increasing values in CAF-VH groups in the L18 condition (*p* = 0.064) and L6 condition (*p* = 0.52) compared to L12 rats, and even a higher level in L6-CAF-GSPE compared to the L12-CAF-GSPE group (*p* = 0.013). The effect of the obesogenic diet was also observed on the liver weight as all CAF-VH groups showed an increase in the weight of this organ compared to their lean controls. Moreover, animals subjected to a long photoperiod (L18) and treated with GSPE showed a decrease in liver weight compared to its CAF-VH group (*p* = 0.01). 

### 2.4. Exposure to Either Long or Short Photoperiods Alters the Expression of Clock Genes in the Livers of Obese Rats

**Figure 4:**
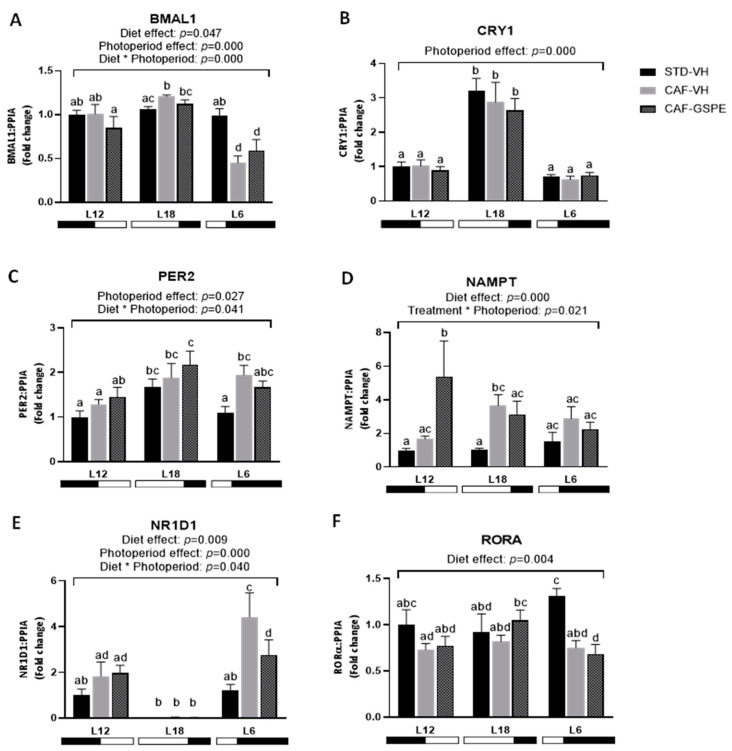
mRNA expression levels of clock genes in the liver of Fischer 344 STD and CAF-fed rats exposed to L12, L18, or L6 photoperiods (n = 8) (**A**–**F**). The results are presented as the mean ± S.E.M. One- and two-way ANOVA following LSD post hoc tests were performed to compare the values between groups, and significant differences (*p* ≤ 0.05) were represented with different letters (a, b, c, d).

The hepatic mRNA expressions of clock genes were analyzed as it is well known that metabolism is regulated not only but largely by circadian rhythms [30]. As is shown in Figure 4, on the one hand, apart from the effect of GSPE consumption in increasing Nicotinamide phosphoribosyl transferase (*Nampt*) gene expression, no further changes in clock genes expressions were seen between groups exposed to L12 photoperiod. On the other hand, CAF-fed animals exposed to long or short photoperiods showed differences in the expression of some clock genes compared to their STD-VH controls. In this regard, expression of the brain and muscle Arnt-like protein-1 (*Bmal1*) gene was higher in L18-CAF-VH (*p* = 0.001), but not in L18-CAF-GSPE rats, compared to L18-STD-VH. In animals exposed to short photoperiods, the CAF diet caused a strong downregulation of the expression of this gene that could not be restored by GSPE treatment. No differences were seen in the expression of the Cryptochrome circadian clock 1 (*Cry1*) gene between groups within each photoperiod, but animals exposed to L18 exhibited a two hundred percent increase in its expression compared to animals exposed to L12 and L6. Regarding expression levels of Period circadian clock 2 (*Per2*), significant differences were seen only in animals exposed to L6, with a significant rise in CAF-VH (*p* = 0.008) but not in CAF-GSPE compared to their STD-VH control. Expression of the nuclear receptor subfamily 1 group D member 1 gene (*Nr1d1*) was totally repressed in animals exposed to long photoperiod (L18). Under short-day conditions, CAF diet caused an upregulation in the expression of this gene compared to its lean STD control (*p* = 0.001), while treatment with GSPE was able to reduce these levels compared to CAF-VH (*p* = 0.025). With *Bmal1* activator, the RAR-related orphan receptor alpha (*Rorα)* gene expression was downregulated in CAF-fed animals subjected to short photoperiod (L6) compared to L6-STD-VH control. 

### 2.5. The Effect of GSPE on the Expression of Lipid- and Glucose-Related Genes in the Liver Is Influenced by CAF Dietary Intake and Chronic Exposure to Different Photoperiods

**Figure 5:**
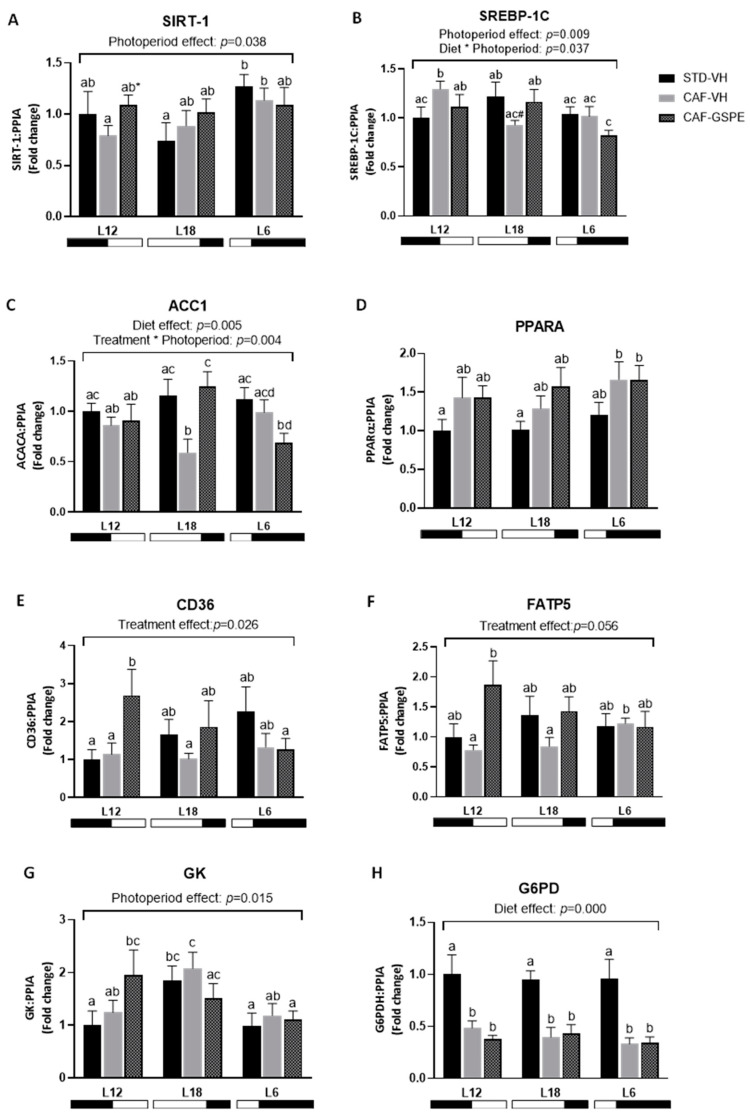
mRNA expression levels of liver lipid and glucose metabolism in Fischer 344 STD and CAF-fed rats exposed to L12, L18, or L6 photoperiods (n = 8) (**A**–**H**). The results are presented as the mean ± S.E.M. One- and two-way ANOVA following LSD post hoc tests were performed to compare the values between groups, and significant differences (*p* ≤ 0.05) were represented with different letters (a, b, c, d). A * indicates tendency between L12-CAF-VH and L12-CAF-GSPE groups (*p* = 0.061). A # indicates tendency between L18-STD-VH and L18-CAF-VH groups (*p* = 0.056).

The mRNA levels of key genes involved in lipid and glucose metabolism were analyzed to evaluate the impact of GSPE treatment on liver metabolism under different photoperiods (Figure 5). In this regard, GSPE showed a trend to increase mRNA levels of Sirtuin 1 (*Sirt1*) compared to CAF-VH group in animals subjected to standard photoperiod (L12) (*p* = 0.061). The transcription factor sterol regulatory element binding protein-1c (SREBP-1c) primarily regulates the expression of genes involved in de *novo* lipogenesis and triglyceride synthesis, including acetyl-CoA carboxylase (*Acacα*). The mRNA levels of SREBP-1c varied according to diet and photoperiod, since the intake of CAF diet increased its expression in L12-CAF-VH but not in L12-GSPE rats compared to its control (*p* = 0.04), whereas tended to decrease its levels in L18-CAF-VH but not in L18-CAF-GSPE animals compared to L18-STD-VH control (*p* = 0.056). Similar results were observed in L18-CAF-VH animals with a significant downregulation of *Acacα* gene expression compared to its lean control group (*p* = 0.004). Moreover, GSPE consumption was able to reverse this downregulation in the expression of *Acacα* gene in these animals that were exposed to long photoperiod compared to L18-CAF-VH *(p* = 0.001). mRNA levels of fatty acid translocase cluster of differentiation 36 (*Cd36)* and fatty acid transport protein 5 (*Fatp5*) were increased in animals treated with GSPE under L12 photoperiod compared to L12-CAF-VH rats (*p* = 0.021 and 0.030, respectively). An effect of photoperiod on the expression of the glucokinase (*Gk*) gene was observed, since its levels were higher in L18 VH animals compared to L12 and L6 VH rats. Regarding glucose-6-phosphate dehydrogenase (*G6pd*) a strong downregulation caused by CAF diet was observed in the expression of this gene that GSPE was not able to revert in any of the three photoperiods. Any differential expression of peroxisome proliferator-activated receptor alpha (*PPARα*) was observed among groups within each photoperiod.

A total of 66 metabolites were evaluated for different photoperiods to study the effect on the serum metabolome of diet (STD and CAF) and treatment (VH and GSPE). The results of the serum metabolome were obtained using GC-qTOP approaches. 

Table 2 summarizes the statistical and predictive analysis of the serum metabolome for L12 groups. Regarding diet effect, 21 out of 66 metabolites were significantly altered between STD-VH and CAF-VH groups after the adjustment of the statistical test. Regarding treatment effect, 4 metabolites were significantly different between CAF-VH and CAF-GSPE groups (proline, 2-hydroxyisobutyric acid, 4-hydroxyproline and 3-Phosphoglyceric acid). In the case of proline, 4-hydroxyproline, and 3-Phosphoglyceric acid, GSPE counteracted the increase produced by the CAF diet effect to levels that were similar to the STD diet ones.

Focusing on the effect of diet in the L18 condition, 24 out of 66 metabolites were significantly altered between STD-VH and CAF-VH groups after the adjustment of the statistical test (Table 3). While CAF-VH and CAF-GSPE animals presented differences in 4 metabolites (leucine, isoleucine, valine and methionine). These altered amino acids suggest some effect of GSPE in the long day period. 

Regarding the L6 photoperiod, Table 4 summarizes the statistical and predictive analysis of the serum metabolome for L6 groups. The outcome showed 16 out of 66 metabolites were significantly altered between STD-VH and CAF-VH groups after the adjustment of the statistical test. The effect of treatment was observed between CAF-VH and CAF-GSPE animals, who presented differences in eight metabolites (d-fructose, 3-hydroxisovaleric acid, 3-hydroxytutiric acid, glycolytic acid, oxalic acid, hippuric acid, aspartic acid, and 2-hydroxybutyric acid).

**Figure 6:**
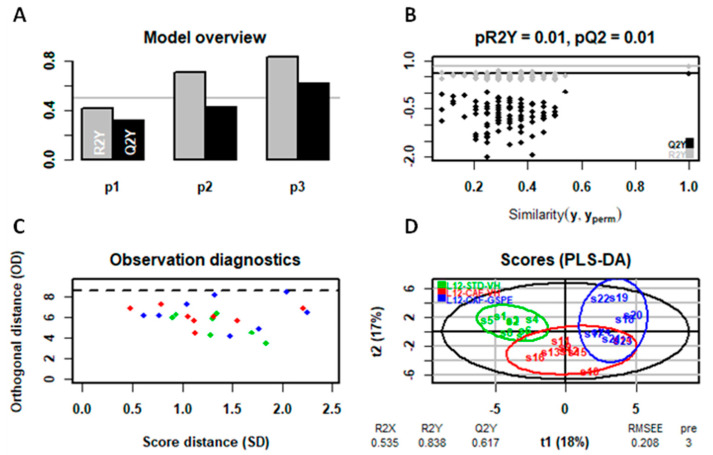
PLS-DA model of the L12 photoperiod (liver metabolome) (**A**–**D**). Top left: inertia barplot: the graphic here suggests that 3 components may be sufficient to capture most of the inertia; Top right: significance diagnostic: the R2Y and Q2Y of the model are compared with the corresponding values obtained after random permutation of the y response; Bottom left: outlier diagnostics; Bottom right: x-score plot, the number of components and the cumulative R2X, R2Y, and Q2Y are indicated below the plot.

**Figure 7:**
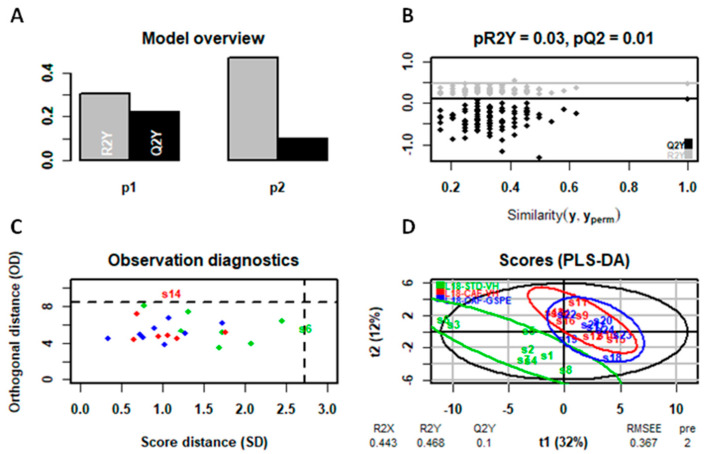
PLS-DA model of the L18 photoperiod (liver metabolome) (**A**–**D**). Top left: inertia barplot: the graphic here suggests that 2 components may be sufficient to capture most of the inertia; Top right: significance diagnostic: the R2Y and Q2Y of the model are compared with the corresponding values obtained after random permutation of the y response; Bottom left: outlier diagnostics; Bottom right: x-score plot, the number of components and the cumulative R2X, R2Y, and Q2Y are indicated below the plot.

**Figure 8:**
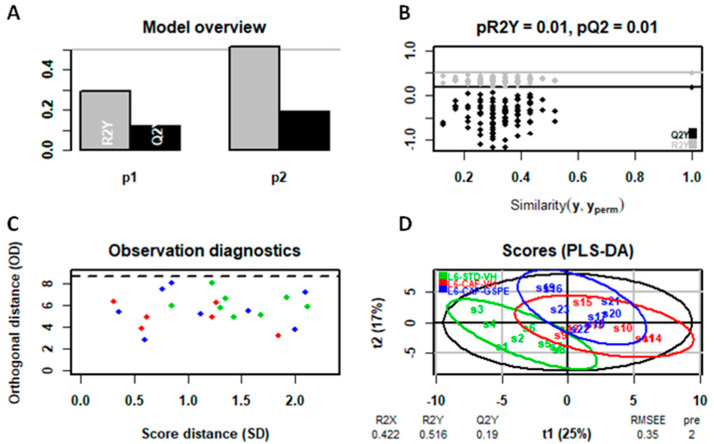
PLS-DA model of the L6 photoperiod (liver metabolome) (**A**–**D**). Top left: inertia barplot: the graphic here suggests that 2 components may be sufficient to capture most of the inertia; Top right: significance diagnostic: the R2Y and Q2Y of the model are compared with the corresponding values obtained after random permutation of the y response; Bottom left: outlier diagnostics; Bottom right: x-score plot, the number of components and the cumulative R2X, R2Y, and Q2Y are indicated below the plot.

The predictive analysis, despite the low clustering in the PCA (Figures S1, S3 and S5), presented differences in the PLS-DA model (specifically in liver metabolome) allowing the differentiation among groups (Figure 6, Figure 7 and Figure 8). In this regard, as illustrated in the figures below, the liver metabolome of L12-photoperiod animals showed the highest discrimination output compared to those in L18 and L6 conditions. This result correlates with the previously observed weight loss, indicating that the standard 12 h light/dark cycle enhances the metabolic benefits of GSPE, leading to more significant improvements in liver metabolism and weight reduction.

## 3. Discussion

Obesity is primarily caused by the combination of an excessive caloric intake and a lack of physical activity. This imbalance between caloric consumption and energy expenditure leads to ectopic fat accumulation in organs such as the liver, pancreas, skeletal muscle, and heart, causing severe metabolic disorders including MS and NAFLD. In addition, several studies suggest that changes in the photoperiod may contribute to increased risk factors of obesity and related metabolic disturbances [9,31]. In this sense, these studies reveal an increase in body weight and disruption of lipid and glucose metabolism in animals exposed to long and especially short photoperiods. Accordingly, in our study, we observed that changes in the photoperiod significantly impacted the metabolism of STD-VH rats. Specifically, rats exposed to short photoperiods (L6) showed a higher body weight gain compared to those in standard (L12) and long photoperiods (L18). Furthermore, lipid parameters such as cholesterol and triglycerides, as well as glucose levels, were also influenced by photoperiod changes. For instance, cholesterol levels were higher in L6 and L18 photoperiods compared to the L12 photoperiod. These findings highlight the intrinsic effect of photoperiod on metabolic regulation. On the other hand, increasing evidence has demonstrated the health benefits associated with obesity of polyphenols, and particularly, GSPE consumption. However, the influence of seasonal variations in day length on the beneficial effects of GSPE consumption on MS and NAFLD has not yet been examined. In this regard, to the best of our knowledge, this study demonstrates for the first time how changes in photoperiod significantly influence the molecular effects of GSPE on metabolic homeostasis, and especially on lipid and glucose metabolism in the liver of obese rats.

The beneficial GSPE effect was initially evidenced by the significant reduction in body weight gain of CAF-fed rats due to GSPE treatment only in the L12 conditions, showing a trend in L18, but no body weight reduction effect was observed in the L6 condition. In agreement with these results, rats fed a CAF diet and subjected to L12 showed an improvement in the serum profile in terms of glucose, cholesterol and triglyceride parameters when treated with GSPE. Despite the absence of any weight loss effect in the L6 photoperiod, GSPE treatment was also able to reduce the levels of triglycerides and to attenuate the increase in glucose levels due to the CAF diet. Conversely, the tendency of lowering weight with GSPE treatment at L18 was not reflected in any amelioration of the previous serum parameters. This could be likely related to the increase in testosterone levels in L18-CAF-GSPE animals as it has been shown that this hormone has a major influence on body fat composition, and its deficiency has been associated with increased central adiposity [32]. Our results are consistent with previous studies showing an improvement in serum lipid and glycemic profiles due to GSPE treatment. Interestingly, these experiments were carried out under standard conditions of 12 h light–12 h darkness, where a better response to GSPE treatment in these parameters was also observed [33,34,35,36].

The liver plays a key role in controlling lipid homeostasis [37]. Interestingly, our study showed that liver lipid levels of CAF-fed rats were greatly influenced by photoperiod exposure. In this sense, exposure to long photoperiods increased levels of cholesterol, whereas triglyceride and phospholipid levels were raised in both long (L18) and short photoperiods (L6) compared to their L12 counterparts. Therefore, these findings confirm previous results showing a strong regulation of lipid levels by photoperiod, increasing its levels in animals fed an obesogenic diet and exposed to long and short photoperiod [38], which can be related to changes in energy expenditure in response to photoperiod. It was also observed that animals fed a CAF diet and exposed to L12 photoperiod showed an increased energy expenditure compared to STD counterparts, which was lost when they were switched to L18 or L6 photoperiods [28]. 

Although no differences in the amount of lipids between CAF-VH and CAF-GSPE were observed, chronic consumption of GSPE was able to reduce liver weight of rats exposed to L18, which could be related with the increase in testosterone levels observed in L18-CAF-GSPE animals, as lower levels of this hormone have been associated with NAFLD [39,40]. 

The bidirectional crosstalk between circadian rhythms and metabolism is evident in the generation of metabolic output pathways by the clock’s transcriptional network, as well as in the mutual ways in which metabolic pathways reprogram the clock [41]. In this regard, L12-GSPE animals showed increased expression of the clock-controlled gene *Nampt* which was correlated with the increase in the expression of *Sirt1* by GSPE in these animals. *Nampt* plays a pivotal role in the regulation of energy homeostasis as it encodes the rate-limiting enzyme responsible for NAD+ biosynthesis, whose intracellular levels determine the expression of *Sirt1* [42]. The NAD+-dependent deacetylase *Sirt1* is a key metabolic gene involved in the regulation of various metabolic processes such as gluconeogenesis, glycolysis, insulin sensitivity, fatty acid oxidation, and cholesterol metabolism in the liver [43]. It has been suggested that the activation of *Sirt1* by polyphenols is beneficial for the regulation of metabolism [44]. In this regard, Aragonés and colleagues reported that GSPE consumption was able to modulate NAD+ levels in the liver and showed that levels of *Sirt1* were increased due to GSPE treatment [45]. Therefore, the modulation of NAD+ homeostasis by GSPE consumption enables these natural compounds to regulate many metabolic processes in the liver. Nevertheless, based on our present results, such modulation appears to be influenced by photoperiod exposure as it was only observed under standard conditions (L12). Furthermore, several studies highlight *Sirt1* and NAD+ levels as promising targets for treating metabolic and cardiovascular diseases [46,47,48].

Furthermore, a human clinical trial in which patients were chronically administered a combined treatment of niacin, a NAD+ precursor, and clofibrate, a *PPPARα* inductor, showed improved levels of serum cholesterol and triglycerides [49]. Another study observed similar results regarding the improvement in serum lipids by niacin treatment [50]. This supports our finding as we observed lower levels of triglycerides and cholesterol in L12-CAF-GSPE rats. In addition, it has been reported that mice with Sirt1 catalytic activity ablation that were fed a high fat diet (HFD) exhibited an increase in SREBP-1c levels and a decreased phosphorylation of AMPK in the liver, promoting NAFLD [51], which is consistent with the results obtained for SREBP-1c in L12 CAF-fed rats. 

L12-CAF-GSPE rats also showed an increase in the expression of *Cd36* and *Fatp5*. It has been demonstrated that hepatic overexpression of *Cd36* in the liver improves glycogen homeostasis and alleviates liver steatosis induced by a HFD in mice [52]. Moreover, expression of *Fatp5* in the liver of NAFLD patients was found to be inversely correlated with the hallmarks of histological progression, such as ballooning and fibrosis [53]. Therefore, *Cd36* and *Fatp5* may act as hepatic “metabolic gatekeepers” protecting the liver under lipid overload and metabolic stress. 

On the other hand, the exposure to short or long photoperiods in CAF-fed rats clearly altered clock gene expression in the liver at a single time point. In this sense, L6-CAF-VH rats exhibited a downregulation of mRNA levels of *Bmal1* and its activator *Rorα*, compared to the healthy control. In addition, the CAF diet increased the expression of the clock genes *Per2* and *Nr1d1*, also known as *Rev-erbα*, while GSPE treated animals not only did not show any significant increase in *Per2* expression compared to the lean control, but also decreased the mRNA levels of *Nr1d1. Rev-erbα* has been suggested as an integrator of circadian rhythms and metabolism [54]. Indeed, it has been shown that liver-specific overexpression of *Rev-erbα* disrupts the molecular clock of the liver, dampening hepatic circadian transcriptional variation to a large extent (nearly 90%) [55]. In addition, *Rev-erbα* is involved in lipid and bile acid homeostasis, and mice lacking this clock gene display dyslipidemia characterized by increased triglyceride levels [56]. This goes in line with our results as we observed a decrease in serum triglyceride levels in L6-GSPE-treated rats. 

In animals exposed to a long photoperiod (L18) and fed a CAF diet, there was a notable increase in Bmal1 expression. However, this significant upregulation was absent in L18-CAF-GSPE animals compared to the lean control group. Previous studies have elucidated the pivotal role of Bmal1 in orchestrating the de novo synthesis of lipids in the liver, primarily through the activation of AKT [57]. Moreover, research utilizing liver-specific Bmal1 knockout mice subjected to ethanol-fed conditions revealed a concomitant inhibition of both de novo lipogenesis and fatty acid oxidation pathways, culminating in hepatic steatosis. Notably, this adverse effect was mitigated by the administration of synthetic PPARα ligands, indicating a potential therapeutic avenue [58]. Consistent with these findings, our study observed a downregulation in the mRNA levels of key lipogenic genes, such as SREBP-1c and its direct target Acacα, which encodes the rate-limiting enzyme in de novo fatty acid synthesis, in L18-CAF-VH animals compared to their control counterparts. Remarkably, consumption of GSPE (grape seed proanthocyanidin extract) was efficacious in restoring the dysregulated expression of these lipogenic genes in animals exposed to a long photoperiod. Furthermore, although the levels of PPARα did not exhibit a significant increase in L18-CAF-GSPE rats, it appeared that GSPE treatment tended towards enhancing its expression. These findings collectively underscore the potential therapeutic efficacy of GSPE in ameliorating metabolic dysregulation associated with prolonged photoperiod exposure and CAF diet consumption, potentially through modulation of Bmal1 and PPARα pathways.

Since the last decade, 24 h rhythms have been described in circulating and tissue metabolites levels varying in concentration in a daily manner [59,60,61,62]. Therefore, metabolites are also expected to vary throughout the year, in a season-dependent manner [63,64,65]. Alterations in the rhythmic expression of serum metabolites were reported due to shift work [66], and disturbances in daily rhythms of hepatic metabolites have been observed in CAF-fed rats [67]. In this sense, we found significant alterations of several serum and hepatic metabolites in CAF-fed obese rats, which vary depending on the photoperiod, as well as the effects of GSPE on these metabolites. In L12 CAF-fed animals, GSPE was able to counteract the increase on serum proline, 4-hydroxyproline, and 3-Phosphoglyceric acid (3PG), caused by the obesogenic diet intake. Proline and 4-hydroxyproline (produced by hydroxylation of the amino acid proline), play a key role in whole-body homeostasis, being essential for metabolism as they are involved in the synthesis and structure of proteins, arginine and glutamate synthesis, and collagen production, as well as in antioxidant reactions, wound healing, and immune responses [68]. The 3PG compound is the acid conjugate of glycerate 3-phosphate. Glycerate is a biochemically important metabolic intermediate in glycolysis. The 3PG compound is also involved in gluconeogenesis, glycerol, lipid metabolism, glycine, serine, and threonine metabolism, playing a key role in several metabolic pathways [69,70,71].

Interestingly, one of the most remarkable effects of GSPE was the increase in serum levels of methionine and the three essential branched-chain amino acids (BCAAs) leucine, isoleucine, and valine, which were decreased due to CAF-diet intake. Methionine is an essential amino acid involved in anabolic metabolism and the reduction of free radicals and has been found to be decreased in MS patients [72] and in obese children [73]. Moreover, disorders of BCAAs have a huge impact on metabolism, as they have been found to be associated with several metabolic diseases [74]. The largest differences in serum metabolite concentrations were observed between CAF-VH and CAF-GSPE in rats subjected to short photoperiod (L6). Among them, 2-hydroxybutyric acid, also known as alpha-hydroxybutyrate (α-HB), displayed a 1.6-fold increase in CAF-GSPE animals compared to CAF-VH. It has been observed that α-HB could act as a potential functional metabolite reversing HFD-induced metabolic changes, as, for example, the reduction of serum triglyceride levels, which is consistent with the results obtained in L6-CAF-GSPE and suggest that this metabolite could be involved, at least partially, in this reduction [75]. Similar results have been observed regarding the levels of 3-Hydroxybutyric acid, also named beta-hydroxybutyrate (β-HB), and of 3-hydroxyisovaleric which have been reported as biomarkers in the diagnosis of diabetic ketoacidosis, a complication of diabetes mellitus [76,77]. In addition, lower levels of β-HB have been observed in the plasma of obese patients [78]. GSPE also raised levels of oxalic acid which has been suggested as a novel treatment for obesity, since it proved to be a highly active lipase inhibitor [79].

Increased fructose concentrations in the blood of patients with T2DM has also been observed [80,81]. Remarkably, GSPE was able to reduce serum D-fructose levels in L6 CAF-fed animals, restoring them to values like those observed in the healthy control. 

This study demonstrates that the effects of grape seed proanthocyanidin extract (GSPE) on the liver and whole-body metabolism of obese rats vary with seasonal changes in day length (photoperiods). GSPE showed the most pronounced metabolic benefits under standard 12 h light/dark conditions, including reductions in body weight gain, cholesterol, and triglycerides, as well as improvements in gene expression and metabolite levels. Under long-day and short-day conditions, GSPE also provided metabolic benefits, though the specific effects differed. 

## 4. Materials and Methods

### 4.1. Animal Handling

**Figure 9:**
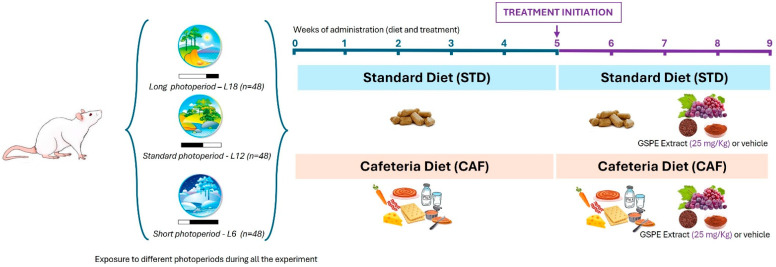
Experimental design summary. Seventy-two 12-week-old male F344 rats were housed in pairs and subjected to three different light schedules over 9 weeks to mimic seasonal day lengths: standard photoperiod L12 (12 h light, 12 h dark), long day photoperiod L18 (18 h light, 6 h dark), and short day photoperiod L6 (6 h light, 18 h dark). Following a 4-day adaptation period with an STD diet (20% protein, 8% fat, 72% carbohydrates), rats were randomly divided into groups based on their photoperiod and diet, with 8 rats per photoperiod fed the STD diet and 16 fed a CAF diet (14% protein, 35% fat, 76% carbohydrates). During a 5-week pre-treatment, CAF-fed rats received biscuits, cheese, pâté, bacon, coiled puff pastry, feed, carrots, and sweetened milk. In the subsequent 4-week treatment period, STD-fed rats received condensed milk as a vehicle (VH), while CAF-fed rats were divided into two groups: one treated with VH and the other with 25 mg/kg GSPE. The rats had free access to water, and their body weight was recorded weekly. After 9 weeks, the rats were fasted for 3 h and sacrificed by decapitation under anesthesia. Blood and liver samples were collected and stored for further analysis.

Seventy-two 12-week-old male F344 rats (Charles River Laboratories, Barcelona, Spain) were housed in pairs in cages at 22 °C and 55% humidity, and subjected to three different light schedules during 9 weeks to mimic seasonal day lengths: normal day or standard photoperiod L12 (*n* = 24, 12 h light and 12 h darkness), long day photoperiod L18 (*n* = 24, 18 h and 6 h darkness), and short day photoperiod L6 (*n* = 24, 6 h light and 18 h darkness). A 4-day adaptation period was carried out where rats were fed with STD diet ad libitum. The STD composition was 20% protein, 8% fat, and 72% carbohydrates (Panlab, Barcelona, Spain). Within each photoperiod rats were randomly divided into 2 groups depending on the diet; 8 rats were fed with STD and 16 rats with CAF diet during a 5-week pre-treatment. The CAF diet consisted of biscuits with cheese and pâté, bacon, coiled puff pastry from Mallorca (Spain), feed, carrots, and sweetened milk (22% sucrose *w*/*v*). CAF composition was 14% protein, 35% fat, and 76% carbohydrates. The treatment period started on the 5th week, lasted four weeks, and was daily orally administered at ZT0 using a syringe. Rats continued with the diet they were fed during the pre-treatment period. All STD-fed rats were treated with condensed milk, named vehicle (VH). Within each photoperiod CAF-fed rats were divided into two groups; 8 were treated with the VH, and 8 with 25 mg/kg GSPE (Les Dérivés Résiniques et Terpéniques, Dax, France) diluted 1/5 in condensed milk. GSPE was composed of catechin (58 μmol/g), dimeric procyanidins (250 μmol/g), epicatechin (52 μmol/g), epigallocatechin (5.50 μmol/g), epicatechin gallate (89 μmol/g), epigallocatechin gallate (1.40 μmol/g), hexameric procyanidins (0.38 μmol/g), pentameric procyanidins (0.73 μmol/g), tetrameric procyanidins (8.8 μmol/g), and trimeric procyanidins (1568 μmol/g) [82]. During the entire study, rats had free access to water, and body weight was weekly recorded. After 9 weeks, animals were fasted for 3 h and then sacrificed by decapitation at 9 am (one hour after light was turned on; ZT1) under anesthesia (sodium pentobarbital, 50 mg/kg body weight). Blood was collected, and serum was obtained by centrifugation (15.000× *g*, 10 min, 4 °C) and stored at −80 °C until analysis. The liver was rapidly weighed, frozen in liquid nitrogen, and stored at −80 °C for further analysis. A summary of the experimental design can be found in Figure 9.

The Animal Ethics Committee of the Universitat Rovira i Virgili (Tarragona, Spain) approved all procedures (reference number 9495 by Generalitat de Catalunya). All the above-mentioned experiments were performed as authorized by the European Directive 86/609/CEE and Royal Decree 223/1988 of the Spanish Ministry of Agriculture, Fisheries and Food, Madrid, Spain.

### 4.2. Serum Analysis

Circulating levels of glucose, total cholesterol, and triglycerides (QCA, Barcelona, Spain) were analyzed by colorimetric enzymatic assay kits according to the manufacturer’s instructions. Serum levels of insulin were measured using a mouse/rat-specific immunometric sandwich enzyme-linked immunosorbent assay (ELISA) kit purchased from Millipore Ibérica (Madrid, Spain). The homeostasis model assessment-estimated insulin resistance (HOMA-IR) index was calculated from insulin and glucose serum levels.

### 4.3. Gene Expression Analysis

Total RNA from the liver was extracted and quantified as previously described [83]. The cDNA was obtained by a reverse transcription of the RNA extracted using a High-Capacity Complementary DNA Reverse Transcription Kit (Thermo Fisher, Madrid, Spain). The quantitative polymerase chain reactions (qPCRs) were performed in 384-well plates in a 7900HT Fast Real-Time PCR (Thermo Fisher, Madrid, Spain) using iTaq™ Universal SYBR^®^ Green Supermix (Bio-Rad, Barcelona, Spain). The thermal cycle used in all qPCRs was 30 s at 90 °C and 40 cycles of 15 s at 95 °C and 1 min at 60 °C. All liver genes were normalized by the housekeeping gene peptidylprolyl Isomerase A (Ppia). The primers used for each gene were obtained from Biomers (Ulm, Germany) (Supplementary Table S1). The relative expression of each gene was calculated according to the Pfaffl method (2001) [84] and normalized using the control group L12-STD-VH. A dilution series of cDNA diluted 1, 10, 100, and 1000 times was run in each plate to provide a standard curve which was used to calculate primer efficiency to ensure efficiency between 1.8 and 2.

### 4.4. Liver Lipid Profile

Extractions of lipids from liver tissue were carried out following the Bligh and Dyer method (1959) [85] and levels of hepatic cholesterol, triglycerides, and phospholipids were measured using a colorimetric kit assay (QCA, Barcelona, Spain).

### 4.5. Serum Hormones Quantification

For determination of melatonin, testosterone, and corticosterone levels, serum samples were thawed at 4 °C. Thus, 50 μL of serum was mixed with 250 μL of methanol containing the internal standard (2 ng/mL). Then, the mixture was vortexed and centrifuged for 5 min at 4 °C and 15,000 rpm. The supernatant was transferred to a new tube and mixed with 700 μL of 0.1% formic acid in water. The sample was loaded to an SPE tube previously conditioned with methanol and 0.1% formic acid in water. The cartridge was washed with 0.1% formic acid in water and dried under high vacuum. The compounds were eluted with 500 μL of methanol. Samples were evaporated in a SpeedVac at 45 °C and reconstituted with 50 μL of water:methanol (60:40, *v*/*v*) and transferred to a glass vial for analysis. Simultaneous detection and quantification of hormone levels were achieved using liquid chromatography coupled to triple quadrupole mass spectrometry (LC-QqQ; from Agilent, Santa Clara, CA, USA).

### 4.6. Metabolome Analysis by GC-MS in Rat Serum and Liver Samples

Metabolomic analysis of the 72 rat serum and liver samples was performed at the Centre for Omic Sciences (COS, Tarragona, Spain) using gas chromatography coupled with quadrupole time-of-flight mass spectrometry (GC-qTOF model 7200, Agilent, Santa Clara, CA, USA). For the serum samples, a protein precipitation extraction was performed by adding eight volumes of methanol:water (8:2)-containing internal standard mixture to the serum samples. For the liver samples, the extraction was performed by adding 400 µL of methanol:water (8:2)-containing internal standard mixture to the liver samples (approx. 10–20 mg). Then, liver samples were homogenized on a bullet blender using a stainless-steel ball. The serum and liver samples were mixed and incubated at 4 °C for 10 min and centrifuged at 19,000× *g*; supernatant was evaporated to dryness before compound derivatization (methoximation and silylation). The derivatized compounds were analyzed by GC-qTOF. Chromatographic separation was based on the Fiehn Method, [32] using a J&W Scientific HP5-MS film capillary column (30 m × 0.25 mm × 0.25 µm, Agilent, Santa Clara, CA, USA) and helium as a carrier gas with an oven program from 60 to 325 °C. Ionization was performed via electronic impact (EI), with electron energy of 70 eV and operating in full-scan mode. Identification of metabolites was performed using commercial standards and by matching their EI mass spectrum and retention time to a metabolomic Fiehn library (from Agilent, Santa Clara, CA, USA), which contains more than 1400 metabolites. After putative identification of metabolites, they were semi-quantified in terms of internal standard response ratio.

### 4.7. Statistical Analysis of Serum and Liver Metabolomic Assays

The results were expressed as the mean ± SEM. Individual comparisons between metabolites were determined by the Kruskal–Wallis H-test, a non-parametric version of ANOVA, due to the variables following the assumption of a non-parametric test. The *p*-value adjustment for multiple comparisons was carried out according to the Benjamin–Hochberg (BH) correction method with a false discovery rate (FDR) of 5% and a Post-hoc Dunn. In parallel, a predictive analysis was conducted to evaluate the prediction power of the oxidative stress model. On the one hand, principal component analysis (PCA), an unsupervised multivariate data projection method, was performed to explore the native variance of the samples. On the other hand, partial least squares discriminant analysis (PLS-DA) was performed to determine the prediction power that supervised multivariate data projection method explores, possible relationships between the observable variables (X), and the predicted variables or target (Y) by regression extensions. The predictive performance of the test set was estimated by the Q2Y parameter calculated through cross-validation. The values of Q2 < 0 suggests a model with no predictive ability, 0 < Q2 < 0.5 suggests some predictive character, and Q2 > 0.5 indicates good predictive ability [86]. The feature importance was calculated through the variable importance in projection (VIP), which reflects both the loading weights for each component and the variability of the response explained by the component. Variables with large VIPs, larger than 1, are the most relevant for the model.

### 4.8. General Statistical Analysis

All data were reported as mean ± standard error of the mean (SEM). Body weight gain, serum and liver biochemical profile, liver weight, and liver gene expression were subjected to Student’s *t* test, and one- and two-way analysis of variance (ANOVA) with the least significant difference test (LSD) for post hoc comparisons using the computer program SPSS version 25 (SPSS Inc., Chicago, IL, USA). Graphics were created using GraphPad Prism 8 software (San Diego, CA, USA). For all analyses, a probability (*p*) value of < 0.05 was considered statistically significant. 

## 5. Conclusions

This study demonstrates that seasonal variations in day length (photoperiods) influence the effects of GSPE consumption on the liver and whole-body metabolism of obese rats. Chronic administration of GSPE in animals fed a CAF diet triggered different responses according to the photoperiod. CAF-fed rats exposed to standard 12 h daylight conditions and treated with GSPE showed a reduction in body weight gain, lower levels of total cholesterol and triglycerides in serum, increased hepatic expression of crucial metabolic genes, and a restorative effect on various serum and liver metabolites altered by the CAF diet. When exposed to long-day conditions (18 h daylight), GSPE treatment tended to decrease body weight gain, increase testosterone levels, reduce liver weight, and restore alterations in hepatic lipogenic gene expression and serum amino acid levels. Under short-day conditions (6 h daylight), GSPE improved serum glucose and triglyceride levels, decreased expression of the hepatic clock gene Nr1d1, and altered serum and hepatic metabolite levels. In conclusion, although GSPE mitigated some metabolic disturbances caused by an obesogenic diet in all photoperiods, it was most effective under the standard 12 h light–dark cycle, highlighting the significant influence of day length variations on its metabolic effects.

Future perspectives derived from this study include conducting more in-depth analyses to investigate the mechanisms of action that determine the photoperiod-dependent efficacy of GSPE. Additionally, the effects of prolonged GSPE consumption, including different photoperiods, and how the treatment would influence metabolic processes associated with circadian rhythms remain unclear. Finally, the results obtained in this study could serve as a basis for developing photoperiod-based dietary recommendations for humans.

## Figures and Tables

**Table 1:** Lipid parameters in the liver of Fischer 344 STD and CAF-fed rats exposed to L12, L18, or L6 photoperiods (n = 8). The results are presented as the mean ± S.E.M. One- and two-way ANOVA following LSD post hoc tests were performed to compare the values between groups, and significant differences (*p* < 0.05) were represented with different letters (a, b, c, d). A * indicates tendency between L12-CAF-VH and L18-CAF-VH groups (*p* = 0.064). A # indicates tendency between L12-CAF-VH and L6-CAF-VH groups *(p* = 0.052). P, photoperiod effect. D, diet effect. D*P, interaction between photoperiod and diet.

Parameters	L12			L18			L6			*2wA*
	STD-VH	CAF-VH	CAF-GSPE	STD-VH	CAF-VH	CAF-GSPE	STD-VH	CAF-VH	CAF-GSPE	
Cholesterol (mg/g)	1.19 ± 0.1 a	1.63 ± 0.1 b	1.84 ± 0.1 bc	1.04 ± 0.1 a	2.05 ± 0.1 c	2.05 ± 0.2 c	1.14 ± 0.1 a	1.78 ± 0.1 bc	2.01 ± 0.3 bc	D. D*P
Triglycerides (mg/g)	2.62 ± 0.2 a	5.18 ± 0.4 b	4.84 ± 0.4 b	3.44 ± 0.3 a	6.75 ± 0.4 c	6.93 ± 0.4 c	3.68 ± 0.3 a	6.90 ± 0.5 c	6.92 ± 0.7 c	*D. P*
Phospholipids (mg/g)	6.82 ± 0.3 ab	5.94 ± 0.3 a	6.36 ± 0.4 a	6.30 ± 0.3 a	7.06 ± 0.4 ab*	7.06 ± 0.3 ab	6.92 ± 0.3 ab	7.16 ± 0.7 ab#	7.89 ± 0.6 b	*P*
Liver weight (g)	14.78 ± 0.5 a	20.3 ± 1.4 bc	18.64 ± 0.6 bc	15.21 ± 0.7 a	20.01 ± 0.5 c	17.53 ± 0.7 b	15.60 ± 0.5 ad	19.23 ± 1.5 bc	17.91 ± 1.3 bcd	*D*

**Table 2:** Summary analysis of the serum metabolome at L12 photoperiod for STD-VH, CAF-VH, and CAF-GSPE groups (n = 8). Relative abundances (AU) of metabolites are presented by the mean ± SEM. The *p*-value represents the adjustment for multiple comparisons carried out according to the Benjamin–Hochberg (BH) correction method with a false discovery rate (FDR) of 5%, and a Post-hoc Dunn. The multivariate analysis is represented by the variable importance in projection (VIP) value of PLS-DA. Variables with large VIPs (VIP > 1), are the most relevant for the model.

Metabolite	STD-VH	CAF-VH	CAF-GSPE	*p*-Value	STD-VH vs. CAF-VH	STD-VH vs. CAF-GSPE	CAF-VH vs. CAF-GSPE	VIP
2-Hydroxyisobutyric acid	1.83 ± 0.11	2.49 ± 0.08	3.09 ± 0.17	<0.001	0.016	<0.001	0.039	1.78
Oleic acid	2.2 ± 0.32	9.61 ± 1.13	6.9 ± 0.67	<0.001	<0.001	0.005	0.129	1.76
Indole-3-propanoic acid	1.7 ± 0.17	0.76 ± 0.18	0.33 ± 0.09	0.001	0.012	<0.001	0.074	1.68
Dodecanoic acid	0.16 ± 0.01	0.32 ± 0.01	0.32 ± 0.03	0.001	0.001	0.001	0.375	1.59
Glycine	5.32 ± 0.13	4.75 ± 0.09	4.6 ± 0.11	0.001	0.006	0.001	0.170	1.41
Serine	10.01 ± 0.32	12.89 ± 0.36	12.63 ± 0.42	0.001	0.001	0.002	0.458	1.57
2-HydroxyButyric acid	0.6 ± 0.04	1.34 ± 0.36	2.08 ± 0.42	0.001	0.013	0.001	0.115	1.34
Hippuric acid	0.43 ± 0.09	0.09 ± 0.02	0.1 ± 0.03	0.002	0.002	0.003	0.500	1.43
Pipecolic acid	0.11 ± 0.01	0.07 ± 0.01	0.06 ± 0.01	0.003	0.005	0.003	0.349	1.45
Heptanoic	0.23 ± 0.01	0.28 ± 0.01	0.32 ± 0.02	0.004	0.023	0.002	0.137	1.37
Glycolic acid	0.1 ± 0.01	0.13 ± 0.00	0.13 ± 0.00	0.006	0.013	0.004	0.251	1.38
Threonic acid	0.82 ± 0.09	1.27 ± 0.13	1.39 ± 0.13	0.009	0.010	0.007	0.362	1.20
Glycerol	0.9 ± 0.16	1.52 ± 0.08	1.44 ± 0.15	0.014	0.009	0.019	0.298	1.28
3-Hydroxyisovaleric acid	0.37 ± 0.02	0.44 ± 0.03	0.49 ± 0.03	0.016	0.050	0.007	0.161	1.27
Proline	23.86 ± 3.30	33.98 ± 2.12	26.32 ± 1.58	0.019	0.018	0.444	0.013	1.18
3-hydroxybutyric acid	8.78 ± 0.69	13.41 ± 1.17	15.42 ± 2.37	0.019	0.011	0.022	0.500	1.10
3-Phosphoglyceric acid	0.02 ± 0.01	0.05 ± 0.00	0.03 ± 0.00	0.024	0.011	0.179	0.050	1.36
Valine	10.23 ± 0.35	9.71 ± 0.31	9 ± 0.30	0.039	0.122	0.016	0.126	1.14
Glucose	29.87 ± 1.18	31.52 ± 1.4	34.74 ± 1.18	0.044	0.179	0.020	0.090	1.14

**Table 3:** Summary analysis of the serum metabolome at L18 photoperiod for STD-VH, CAF-VH, and CAF-GSPE groups (n = 8 animals per group). Relative abundances (AU) of metabolites are presented by the mean ± SEM. The *p*-value represents the adjustment for multiple comparisons carried out according to the Benjamin–Hochberg (BH) correction method with a false discovery rate (FDR) of 5%, and a Post-hoc Dunn. The multivariate analysis is represented by the variable importance in projection (VIP) value of PLS-DA. Variables with large VIPs (VIP > 1) are the most relevant for the model.

Metabolite	STD-VH	CAF-VH	CAF-GSPE	*p*-Value	STD-VH vs. CAF-VH	STD-VH vs. CAF-GSPE	CAF-VH vs. CAF-GSPE	VIP
2-HydroxyButyric acid	0.52 ± 0.02	2.07 ± 0.28	1.23 ± 0.15	<0.001	<0.001	0.006	0.069	1.73
Glycolic acid	0.09 ± 0.00	0.13 ± 0.01	0.12 ± 0.01	<0.001	<0.001	0.003	0.161	1.51
d-Sucrose	0.04 ± 0.01	0.37 ± 0.11	0.66 ± 0.17	0.001	0.005	<0.001	0.153	1.31
Dodecanoic acid	0.17 ± 0.00	0.29 ± 0.04	0.35 ± 0.04	0.001	0.003	0.001	0.274	1.23
Oleic acid	3.15 ± 0.35	6.92 ± 1.06	8.91 ± 0.87	0.001	0.007	<0.001	0.137	1.44
Glycerol	1.07 ± 0.08	1.67 ± 0.11	1.64 ± 0.11	0.001	0.001	0.002	0.336	1.34
Threonic acid	0.68 ± 0.06	1.47 ± 0.24	1.53 ± 0.19	0.001	0.002	0.001	0.336	1.22
Serine	10.17 ± 0.23	13.34 ± 0.70	13.9 ± 0.33	0.001	0.007	0.001	0.161	1.47
2-Hydroxyisobutyric acid	1.78 ± 0.16	3.05 ± 0.19	2.65 ± 0.25	0.002	0.001	0.010	0.161	1.39
Hippuric acid	0.53 ± 0.09	0.1 ± 0.02	0.12 ± 0.03	0.002	0.003	0.003	0.402	1.45
Indole-3-propanoic acid	1.49 ± 0.25	0.38 ± 0.08	0.71 ± 0.31	0.005	0.004	0.007	0.323	1.15
Pyruvic acid	17.86 ± 1.19	24.24 ± 0.80	23.07 ± 1.65	0.006	0.003	0.021	0.179	1.22
Fumaric acid	1.19 ± 0.11	2.22 ± 0.35	2.86 ± 0.53	0.007	0.016	0.004	0.240	1.17
Pipecolic acid	0.11 ± 0.01	0.07 ± 0.01	0.06 ± 0.01	0.010	0.015	0.006	0.298	1.23
Leucine	7.82 ± 0.14	6.94 ± 0.26	8.18 ± 0.30	0.011	0.054	0.122	0.005	1.66
3-Hydroxyisovaleric acid	0.34 ± 0.02	0.42 ± 0.01	0.41 ± 0.02	0.013	0.006	0.033	0.198	1.11
Methionine	3.73 ± 0.09	3.32 ± 0.10	3.61 ± 0.05	0.013	0.006	0.198	0.033	1.48
Malic acid	0.98 ± 0.11	1.49 ± 0.22	2.02 ± 0.33	0.014	0.058	0.006	0.129	1.18
4-hydroxyPhenyllactic acid	23.56 ± 5.77	38.09 ± 5.54	52.31 ± 7.62	0.014	0.058	0.006	0.129	1.22
Valine	9.65 ± 0.22	8.46 ± 0.26	9.67 ± 0.42	0.016	0.010	0.486	0.018	1.41
Oxoproline	122.6 ± 4.81	138.36 ± 4.8	147.59 ± 7.23	0.024	0.042	0.012	0.229	1.10
3-hydroxybutyric acid	10.25 ± 0.86	18.3 ± 2.32	13.99 ± 2.11	0.027	0.011	0.108	0.110	1.18
Isoleucine	5.12 ± 0.13	4.72 ± 0.22	5.6 ± 0.24	0.035	0.129	0.110	0.015	1.48
Phenylalanine	4.25 ± 0.09	3.75 ± 0.12	4.02 ± 0.10	0.038	0.016	0.145	0.103	1.31

**Table 4:** Summary analysis of the serum metabolome at L6 photoperiod for STD-VH, CAF-VH, and CAF-GSPE groups (n = 8 animals per group). Relative abundances (AUs) of metabolites are presented by the mean ± SEM. The *p*-value represents the adjustment for multiple comparisons carried out according to the Benjamin–Hochberg (BH) correction method with a false discovery rate (FDR) of 5%, and a Post-hoc Dunn. The multivariate analysis is represented by the variable importance in projection (VIP) value of PLS-DA. Variables with large VIPs (VIP > 1) are the most relevant for the model.

Metabolite	STD-VH	CAF-VH	CAF-GSPE	*p*-Value	STD-VH vs. CAF-VH	STD-VH vs. CAF-GSPE	CAF-VH vs. CAF-GSPE	VIP
Oleic acid	2.34 ± 0.38	7.92 ± 0.90	5.5 ± 0.47	0.001	<0.001	0.005	0.116	1.77
2-HydroxyButyric acid	0.59 ± 0.04	1.32 ± 0.23	2.16 ± 0.28	0.001	0.297	<0.001	0.046	1.57
Glycolic acid	0.1 ± 0.00	0.11 ± 0.00	0.13 ± 0.00	0.001	0.042	<0.001	0.037	1.64
d-Sucrose	0.02 ± 0	0.4 ± 0.14	0.41 ± 0.17	0.001	0.002	0.001	0.406	1.09
d-Fructose	0.05 ± 0.00	0.13 ± 0.05	0.05 ± 0.00	0.002	0.001	0.290	0.003	1.21
Dodecanoic acid	0.16 ± 0.01	0.27 ± 0.02	0.25 ± 0.01	0.002	0.001	0.005	0.204	1.48
Hippuric acid	0.42 ± 0.08	0.09 ± 0.01	0.05 ± 0.01	0.002	0.050	0.001	0.045	1.61
Serine	9.87 ± 0.34	13.27 ± 0.69	13.4 ± 0.8	0.002	0.002	0.003	0.487	1.43
Pipecolic acid	0.1 ± 0.01	0.07 ± 0.00	0.05 ± 0.01	0.003	0.013	0.002	0.208	1.52
Glycerol	0.81 ± 0.07	1.58 ± 0.16	1.37 ± 0.14	0.006	0.004	0.011	0.243	1.43
Glutamine	0.18 ± 0.02	0.31 ± 0.07	0.39 ± 0.04	0.011	0.096	0.004	0.076	1.20
3-Hydroxyisovaleric acid	0.4 ± 0.03	0.41 ± 0.03	0.51 ± 0.02	0.011	0.484	0.014	0.008	1.40
Indole-3-propanoic acid	1.33 ± 0.25	0.76 ± 0.15	0.44 ± 0.14	0.013	0.063	0.005	0.125	1.28
Glucose	29.83 ± 1.11	34.28 ± 0.95	36.23 ± 2.74	0.014	0.016	0.008	0.464	1.01
Heptanoic	0.24 ± 0.01	0.29 ± 0.02	0.33 ± 0.02	0.016	0.033	0.008	0.238	1.23
3-hydroxybutyric acid	9.75 ± 0.97	11.73 ± 3.25	17.06 ± 1.50	0.020	0.467	0.022	0.014	1.27
Threonine	8.23 ± 0.26	10.06 ± 0.56	9.66 ± 0.55	0.026	0.016	0.028	0.289	1.13
Lactic acid	64.93 ± 2.27	74.75 ± 2.12	81.25 ± 6.33	0.036	0.027	0.027	0.420	1.06
Methionine	3.67 ± 0.10	3.52 ± 0.06	3.34 ± 0.07	0.038	0.183	0.018	0.086	1.13

## Data Availability

The data presented in this study are available on request from the corresponding author. The data are not publicly available due to the lack of a platform to publish them.

## Supplementary Materials

The following supporting information can be downloaded at: https://www.mdpi.com/article/10.3390/ijms25147713/s1.
