# Supplementary material for: Grape Seed Proanthocyanidin Extract Attenuates Cafeteria-Diet-Induced Liver Metabolic Disturbances in Rats: Influence of Photoperiod
Source: Int J Mol Sci. 2024 Jul 14;25(14):7713. doi: 10.3390/ijms25147713 (PMC11276873; doi:10.3390/ijms25147713)
Supplement: Supplementary file 1 [file ijms-25-07713-s001.zip › ijms-3039534-supplementary.pdf]

SUPPLEMENTARY MATERIAL

Table S1. Nucleotide sequences of primers used for real-time quantitative PCR

Gene	Forward primer (5' to 3')	Reverse primer (5' to 3')
Acaca	TGCAGGTATCCCCACTCTTC	TTCTGATTCCCTTCCCTCCT
Bmal1	GTAGATCAGAGGGCGACGGCTA	CTTGTCTGTAAAACTTGCCTGTGAC
Cd36	GTCCTGGCTGTGTTTGGA	GCTCAAAGATGGCTCCATTG
Cry1	TGGAAGGTATGCGTGTCCTC	TCCAGGAGAACCTCCTCACG
Fatp5	CCTGCCAAGCTTCGTGCTAAT	GCTCATGTGATAGGATGGCTGG
G6pd	ACCAGGCATTCAAAACGCAT	CAGTCTCAGGGAAGTGTGGT
Gk	CTGTGAAAGCGTGTCCACTC	GCCCTCCTCTGATTCGATGA
Nampt	CTCTTCACAAGAGACTGCCG	TTCATGGTCTTTCCCCCACG
Nr1d1	ACAGCTGACACCACCCAGATC	CATGGGCATAGGTGAAGATTTCT
Per2	CGGACCTGGCTTCAGTTCAT	AGGATCCAAGAACGGCACAG
Pparα	CGGCGTTGAAAACAAGGAGG	TTGGGTTCCATGATGTCGCA
Ppia	CCAAACACAAATGGTTCCCAGT	ATTCCTGGACCCAAAACGCT
Rora	CCCGATGTCTTCAAATCCTTAGG	TCAGTCAGATGCATAGAACACAAACT C
Sirt1	TTGGCACCGATCCTCGAA	ACAGAAACCCCAGCTCCA
Srebp1c	CCCACCCCCTTACACACC	GCCTGCGGTCTTCATTGT

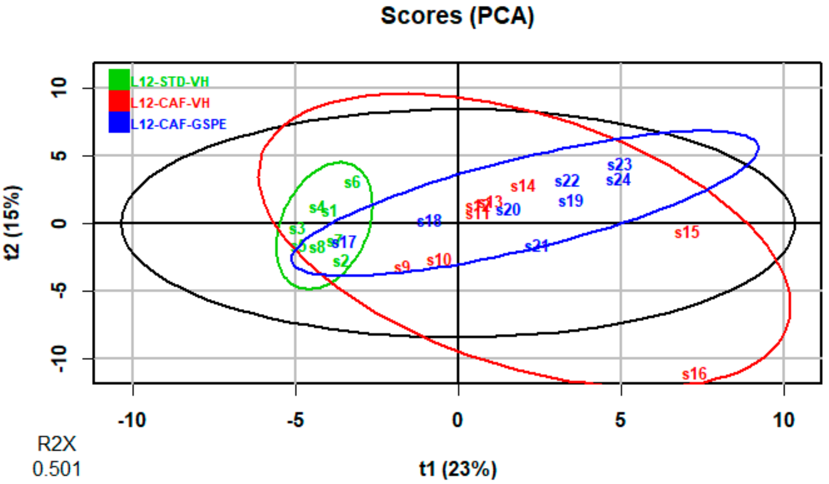

Figure S1. PCA score plot coloured according to groups at L12 photoperiod (serum metabolome).

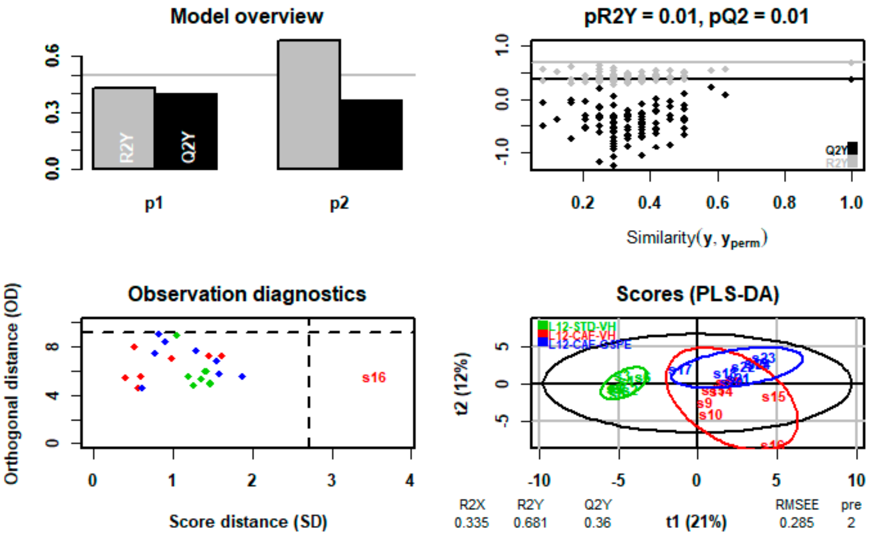

Figure S2. PLS-DA model of the L12 photoperiod (serum metabolome). Top left: inertia barplot: the graphic here suggests that 2 components may be sufficient to capture most of the inertia; Top right: significance diagnostic: the R2Y and Q2Y of the model are compared with the corresponding values obtained after random permutation of the y response; Bottom left: outlier diagnostics; Bottom right: x-score plot: the number of components and the cumulative R2X, R2Y and Q2Y are indicated below the plot.

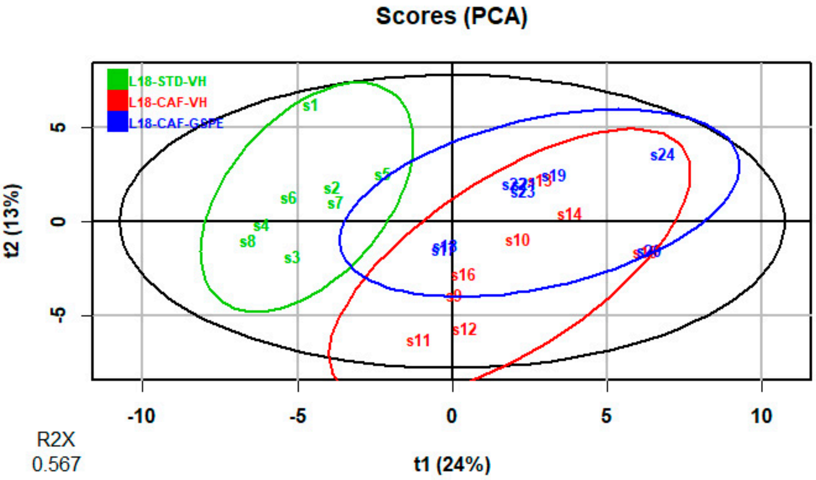

Figure S3. PCA score plot coloured according to groups at L18 photoperiod (serum metabolome).

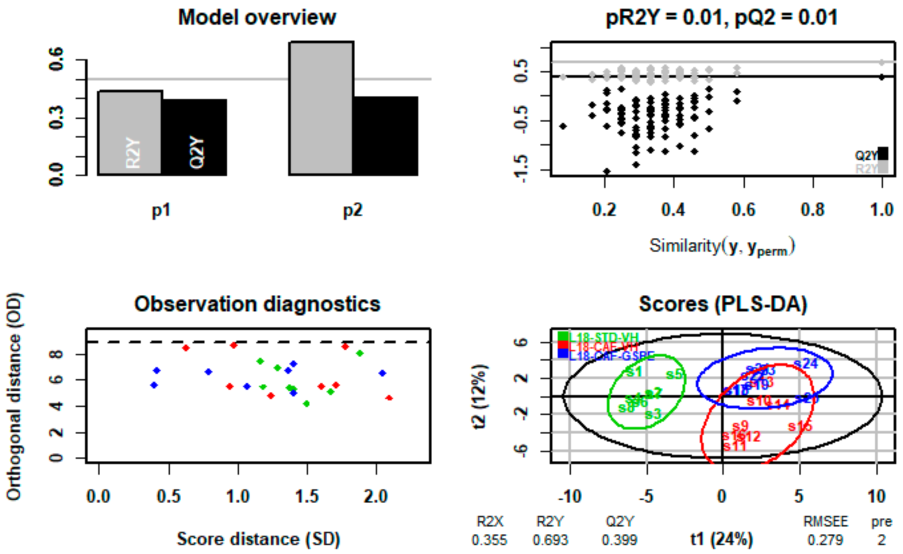

Figure S4. PLS-DA model of the L18 photoperiod (serum metabolome). Top left: inertia barplot: the graphic here suggests that 2 components may be sufficient to capture most of the inertia; Top right: significance diagnostic: the R2Y and Q2Y of the model are compared with the corresponding values obtained after random permutation of the y response; Bottom left: outlier diagnostics; Bottom right: x-score plot: the number of components and the cumulative R2X, R2Y and Q2Y are indicated below the plot.

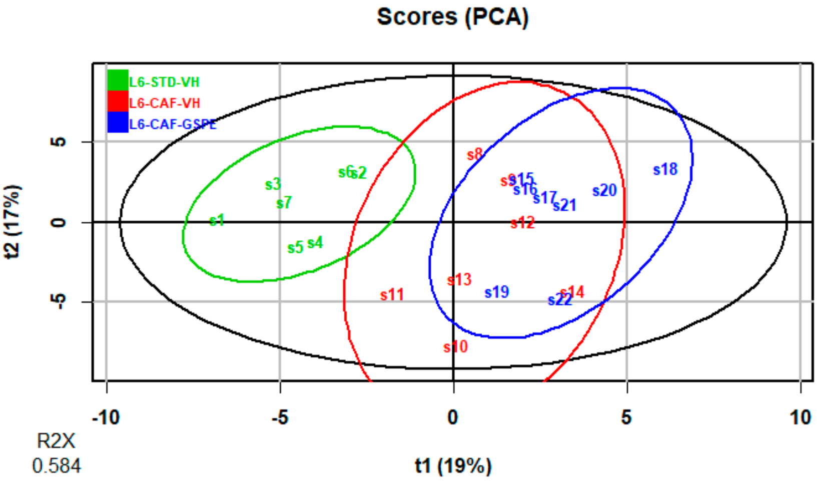

Figure S5. PCA score plot coloured according to groups at L6 photoperiod (serum metabolome).

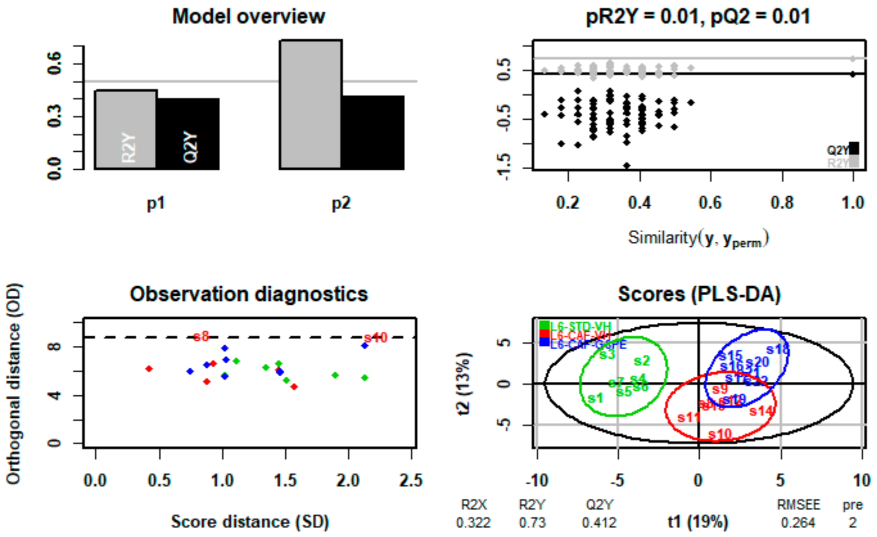

Figure S6. PLS-DA model of the L6 photoperiod (serum metabolome). Top left: inertia barplot: the graphic here suggests that 2 components may be sufficient to capture most of the inertia; Top right: significance diagnostic: the R2Y and Q2Y of the model are compared with the corresponding values obtained after random permutation of the y response; Bottom left: outlier diagnostics; Bottom right: x-score plot: the number of components and the cumulative R2X, R2Y and Q2Y are indicated below the plot.

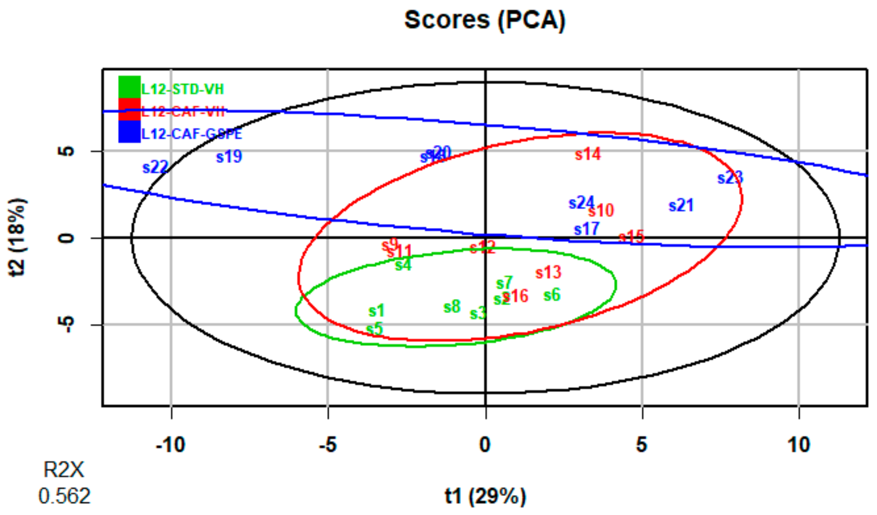

Figure S7. PCA score plot coloured according to groups at L12 photoperiod (liver metabolome).

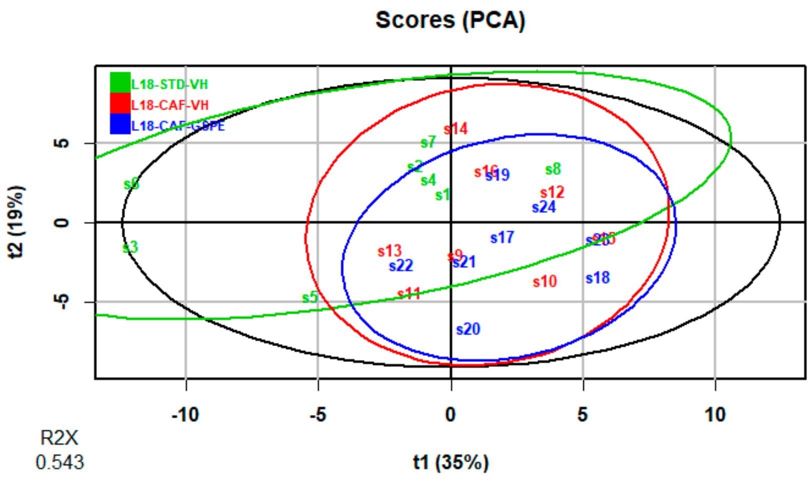

Figure S8. PCA score plot coloured according to groups at L18 photoperiod (liver metabolome).

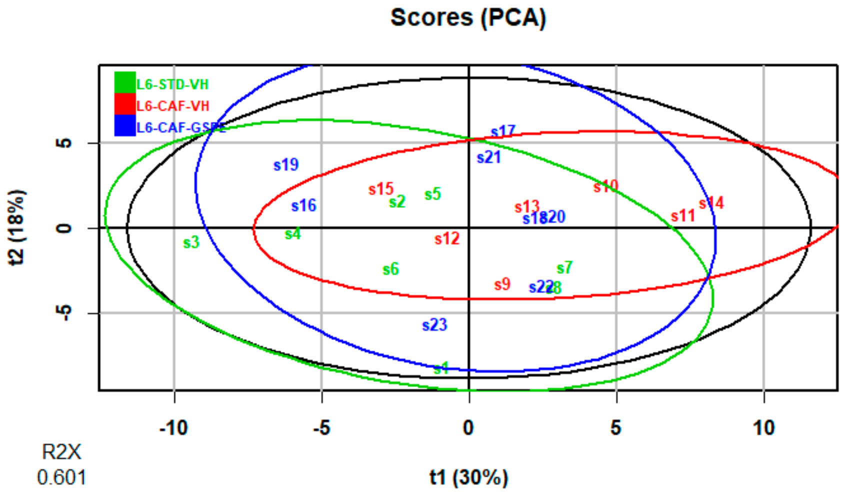

Figure S9. PCA score plot coloured according to groups at L6 photoperiod (liver metabolome).
